# Testing Pollen of Single and Stacked Insect-Resistant Bt-Maize on *In vitro* Reared Honey Bee Larvae

**DOI:** 10.1371/journal.pone.0028174

**Published:** 2011-12-16

**Authors:** Harmen P. Hendriksma, Stephan Härtel, Ingolf Steffan-Dewenter

**Affiliations:** Department of Animal Ecology and Tropical Biology, Biocentre, University of Würzburg, Würzburg, Germany; Ghent University, Belgium

## Abstract

The ecologically and economic important honey bee (*Apis mellifera*) is a key non-target arthropod species in environmental risk assessment (ERA) of genetically modified (GM) crops. Honey bee larvae are directly exposed to transgenic products by the consumption of GM pollen. But most ERA studies only consider responses of adult bees, although Bt-proteins primarily affect the larval phases of target organisms. We adopted an *in vitro* larvae rearing system, to assess lethal and sublethal effects of Bt-pollen consumption in a standardized eco-toxicological bioassay. The effects of pollen from two Bt-maize cultivars, one expressing a single and the other a total of three Bt-proteins, on the survival and prepupae weight of honey bee larvae were analyzed. The control treatments included pollen from three non-transgenic maize varieties and of *Heliconia rostrata*. Three days old larvae were fed the realistic exposure dose of 2 mg pollen within the semi-artificial diet. The larvae were monitored over 120 h, until the prepupal stage, where larvae terminate feeding and growing. Neither single nor stacked Bt-maize pollen showed an adverse effect on larval survival and the prepupal weight. In contrast, feeding of *H. rostrata* pollen caused significant toxic effects. The results of this study indicate that pollen of the tested Bt-varieties does not harm the development of *in vitro* reared *A. mellifera* larvae. To sustain the ecosystem service of pollination, Bt-impact on *A. mellifera* should always be a crucial part of regulatory biosafety assessments. We suggest that our approach of feeding GM pollen on *in vitro* reared honey bee larvae is well suited of becoming a standard bioassay in regulatory risk assessments schemes of GM crops.

## Introduction

Pollinators provide key ecosystem services by maintaining both the biodiversity of wild plants and agricultural productivity [1], [2] at an estimated value of US $217 billion yearly [3]. The most important pollinator species worldwide is the honey bee *Apis mellifera* [4], with populations present in all countries growing genetically modified (GM) crops [5], [6]. Hence, honey bees are a key non-target test species for assessing the potential adverse impacts of GM crops on pollinators [7], [8].

Crops expressing insecticidal proteins derived from the bacterium *Bacillus thuringiensis* (Bt) (Bt-proteins) are among the most widely cultivated GM crops worldwide [6]. A recent meta-analysis showed no adverse effects of Bt-crops on *A. mellifera* [7]. All of the re-analyzed studies tested only the effect of single Bt-proteins. However, one future trend in plant biotechnology is the stacking of multiple resistance traits. An example is Bt-maize SmartStax™, released in 2010 in the USA with six different insect resistance genes for above- and below-ground insect control, with two additional herbicide tolerance genes [6]. Hence, regulatory authorities are in need of up to date test-standards, to guide robust first-Tier laboratory experiments to assess the risks of new GM plants to non-target organisms [9].

Floral pollen is the sole protein source of *A. mellifera* colonies [10] and pollen of a variety of important crops is collected by bee foragers [8]. Adults and larvae of *A. mellifera* are directly exposed to transgenic material via pollen consumption of GM-crops, as planted in mass monocultures. On average, a worker consumes 3.4 to 4.3 mg of pollen per day [10], with colonies accumulating up to 55 kg per year [11]. Bees exposed to Mon810 maize pollen did not transmit quantifiable amounts of the Bt-proteins via their hypopharyngeal glands into the larval food they secrete [12]. Nevertheless, pollen is also straightforwardly added by nurse bees to the larval food [13]. It was reported that larvae consumed 1720–2310 maize pollen grains under semi-field exposure conditions, which is reflecting a worst case maize pollen exposure of 1.52–2.04 mg [14]. In comparison, European butterfly larvae fed with pollen grains from the transgenic maize variety Bt-176 were lethally affected at much lower exposure doses: LD_50_ value of only 8 pollen grains per Diamond-back moth larva, and 32–39 pollen grains for Small tortoiseshells, Peacocks, European corn borers and Cabbage white larvae [15].

Bt-proteins confer plant-protection against herbivorous insects, with immature holometabolous pest insects showing a high susceptibility by a lethal damage to the gut [16]. This considering, especially young honey bee larva are amenable as non-target test organisms for GM crop pollen, because they represent a potentially sensitive life stage. In addition to larvae, young hive bees consume the most pollen within colonies [17], thus young bees are also amenable for precautionary tests on biosafety. Nonetheless, Bt susceptibility in target insect adults is considered limited [18], in comparison to the lethal effects on larval stages [19], [20]. To date, only minor fractions of peer-reviewed pollen feeding studies assess the risks on honey bee larvae [8]. Studies on Bt-pollen feeding to larvae have solely been performed within colonies [7], [21]. In general, studies on the colony level are confounded by environmental influences and by nurse bees which remove the dietary treatments of the larvae. Thus, to be robust, laboratory bioassays need to exclude such uncontrolled factors as far as possible [9]. In this paper, to assess possible lethal and sublethal effects of GM crop pollen on the survival and prepupal weight of individual *A. mellifera* larvae, we adopted a controlled *in vitro* rearing bioassay [22], [23]. The test larvae were exposed by adding fresh Bt-maize pollen directly in their artificial diet. This approach simulates the natural way of pollen consumption, whereby pollen is digested within the gut and Bt-proteins get exposed. Mechanistically, this is of key importance as the lethality among target-organisms is caused by the disruption of the gut epithelium by Bt-protein-receptor interactions [24]. This study fills an important gap in ERA's on bees, as laboratory feeding tests of Bt-pollen on bee larvae are completely lacking.

## Materials and Methods

### Pollen

Multiple pollen types were collected for the *in vitro* pollen feeding experiment (Table 1). Pollen of field grown maize varieties were collected by shaking flowering maize tassels in paper bags. The freshly collected maize pollen was sieved (Ø 0.32 mm). Preceding storage at −80° Celsius, the pollen was dehydrated for 24 hours at room temperature to prevent the grains to burst at freezing.

**Table 1:** Feeding treatments of *in vitro* reared honey bee larvae for the Bt-pollen bioassay.

	Treatment^a^	Plant variety	n Larvae	Colonies	Pollen/2 mg
1	Transgenic maize	Stacked Bt; Mon89034×Mon88017	20	5	1701
2	Transgenic maize	Single Bt; DKc7565	20	5	1750
3	Control maize	Near isogenic line; DKc5340	19	5	1784
4	Control maize	Distant related; DKc4250	20	5	1753
5	Control maize	Unrelated; Benicia	20	5	1722
6	No pollen control	-	12	6	0
7	Positive toxic control	*Heliconia rostrata* (H)	10	5	1600
1,2	Pooled Bt-maize	Transgenic maize (Bt)	40	5	1726
3,4,5	Pooled control maize	Control maize (C)	59	5	1753

a Treatment maize 1 expresses three Bt-proteins encoded by the genes *cry1A.105*, *cry2Ab2* and *cry3Bb1* from *Bacillus thuringiensis* that confer resistance against certain lepidopteran and coleopteran pests and additionally expresses the *CP4 epsps* gene for glyphosate-tolerance. Treatment maize 2 expresses a single lepidopteran specific Bt-toxin encoded by the gene *cry1Ab*. In addition, control treatments, tested plant varieties, number of larvae, colonies and counted pollen grains per 2 mg pollen treatment are indicated.

Pollen of the single transgenic Bt-maize event Mon810 (DKc7565, cultivar Novelis, Monsanto Co.) was collected on July 24^th^ 2008 near Kitzingen (Lower Franconia, Germany). This maize variety expresses Cry1Ab proteins for the control of stemborers such as the European corn borer *Ostrinia nubilalis* (Hübner) (Lepidoptera: Crambidae) at concentrations of 1–97 ng/g fresh weight (fwt) in pollen [25].

Pollen of a stacked Bt-variety was collected in the week of August 4^th^ 2008 near Braunschweig (Germany). This maize variety expresses three genes for insect resistance and one gene for herbicide tolerance and was obtained by a traditional cross of the maize varieties Mon89034 and Mon88017. Line Mon89034 confers resistance to a wide range of butterflies and moths, such as the fall armyworm (*Spodoptera sp.*), the black cutworm (*Agrotis ipsilon*), the european corn borer (*Ostrinia nubilalis*) and the corn earworm (*Helicoverpa zea*) by expression of the Bt-proteins Cry1A.105 at a level of mean 4.24 µg/g (n = 16, fwt in pollen, Sauer and Jehle, pers. comm.) and Cry2Ab2 at a level of mean 1.19 µg/g (n = 16, fwt in pollen, Sauer and Jehle, pers. comm.). Cry1A.105 is a chimeric gene synthesized by combining 4 native Bt-gene domains of *cry1Ab*, *cry1F* and *cry1Ac* [26]. This chimeric protein provides an increased activity against lepidopteran species compared to the original Cry1Ab protein as expressed in Mon810. The other parental line, Mon88017 (DKc5143), confers resistance to coleopteran pests, the Western, Northern and Mexican corn rootworms *Diabrotica spp.* (Coleoptera: Chrysomelidae) by the expression of the Bt-protein Cry3Bb1 at levels of mean 6.95 µg/g (n = 16, fwt in pollen, Sauer and Jehle, pers. comm.) (trademark YieldGard ® Rootworm). Mon88017 also expresses an *Agrobacterium sp.* CP4 derived 5-enolpyruvylshikimate-3-phosphate synthase (CP4 epsps) confering tolerance against glyphosate, the active ingredient of the herbicide Roundup (trademark Roundup Ready®) at an expression level of 170 µg/g (fwt in pollen; www.gmo-compass.org/pdf/regulation/maize/MON89034MON88017_application.pdf).

Stacked Bt-maize pollen and also control pollen of three conventional maize varieties was collected in the week of August 4^th^ 2008 near Braunschweig (Germany). These maize varieties were grown on an experimental field in a randomized block-design with eight replications. Samples were collected from all 30×40 m subplots, pooling the pollen into one representative sample per variety. The non-GM variety DKc5340 (Monsanto Co.) is near-isogenic to the tested stacked Bt-maize variety, DKc4250 (Monsanto Co.) is more distantly related and Benicia (Pioneer HiBred, Johnston, Iowa, USA) is totally unrelated to the stacked event (Table 1).

Pollen of the neotropical plant *Heliconia rostrata* was collected June 23^rd^ 2009 from the greenhouse in the botanical garden of the University of Bayreuth (Upper-Franconia, Germany). The *Heliconia* family is known to have chemical defenses against herbivores [27] and anecdotal brood mortality is known for *Heliconia* foraging honey bee colonies. The pollen of the flowers was collected in a 1.5 ml tube by shaking and scraping pollen from the anthers with a scalpel (45 mg pollen from 41 flowers).

### *In vitro* larvae rearing and treatment applications

The rearing of larvae upon hatchment under laboratory conditions was performed following the protocols by Aupinel et al. and Hendriksma et al. [22], [23]. Six donor honey bee colonies were selected from different Upper-Franconian apiaries, choosing naturally mated non-sibling queens (*Apis mellifera carnica*). By means of an excluder lid, the queens were trapped within their colonies on artificial combs (Nicoplast^©^) (day 1; D1, 25^th^ June 2009). After 91 hours, without grafting manipulation, larvae within plastic queen cups were collected from the combs. Considering a 72 hours development time of the embryos until the hatchment of eggs, the larvae had a mean chronological age of 9:30 hours (D4; min. 0 to max. 19 hours old) and were typically first instars [23].

The subsequent laboratory rearing was performed with larvae in queen-cups mounted in culture plates, placed in a hermetic plexiglass desiccator within an incubator at 35° Celsius. The larvae were fed once a day over D4 to D9 with a 10-, 10- 20-, 30-, 40-, 50-µl semi-artificial diet, respectively [22]. The daily diets were administered with pipettes, adding each new diet into the diet in which individual larvae were floating. Each larva was fed the total amount of 160 µl, since no diet was removed during or after the feeding period. The diet consisted of 50% royal jelly (Le Rucher du Buzard certified organic apiary, Sospel, France) mixed with a 50% aqueous solution. The yeast extract/glucose/fructose proportion in the aqueous solutions was respectively 2/12/12 percent at D4 and D5; 3/15/15 percent at D6; and 4/18/18 percent at D7, D8 and D9 [22]. During larval development, relative humidity in the incubator was kept at 96% using a saturated solution of K_2_SO_4_. Further development upon hatching took place in 80% humidity, maintained using a saturated solution of NaCl. The survival of larvae preceding treatment was 97% (D4 to D6). For more details about the method please see Hendriksma et al. 2011 [23].

For each treatment, a stock solution of 50 mg pollen per 500 µl D6-diet was made. This application is in agreement with empirical findings that the food of larvae contains pollen from the third instar stage onwards (D6) [28], [29]. In this way each 20 µl treatment diet contained a 2 mg pollen dose per larvae [14]. Mean pollen numbers per dose were obtained by 8 sample counts per stock solution using a Neubauer improved counting chamber and a light-microscope (Table 1). The larvae were only once given a dietary pollen dose (D6). Because the larvae did not finish their daily dietary amounts within 24 h, the pollen were consumed over the remaining total exposure time, until the diet was completely finished at the non-feeding days D10/D11. The maize pollen varieties were tested on N = 20 larvae per treatment (5 colonies×4 larvae). *Heliconia* pollen and a no-pollen control treatment were performed on N = 10 and N = 12 larvae respectively (Table 1).

The survival of larvae during the experiment was noted daily, to assess possible lethal effects of Bt-maize pollen during the 120 hours of dietary exposure. By weighing the prepupae after defecation (D11), a potential sublethal effect was monitored. As larvae defecate and molt their intestine at this stage, both the exposure and the potential Bt-protein-receptor based mechanism are physically terminated. Hence, the effective gain in weight can only measured after defecation. Every prepupa was transplanted with soft metal tweezers into a new clean cell on an analytical microbalance to measure the weight to the nearest 0.001 g.

### Statistics

The data were analyzed with mixed models using different packages of the open source statistic software R version 2.11.1 [30]. The identity of the replicate donor colonies was included as a random factor in the models to take the non-independence of larvae from individual colonies into account [23].

Prepupae weights were analyzed with linear mixed effects models using the package *nlme* [31]. The survival dynamics of larvae were analyzed with Cox proportional hazards regression models [32] using the R packages *survival* and *survnnet* [33], [34]. A dynamic survival analysis is not applicable when all individuals of a group survive; in that case a Chi-square analysis was used.

Three test levels were considered. An overall sort-effect was tested over the five maize varieties. All treatments were also tested individually, paired to one another, to indicate sort effects. The significance of *P* values (α = 0.05) of multiple comparison were determined with an α-correction using the sequential Holm-Bonferroni procedure [35]. In case of no detectable difference, the treatment comparisons were summarized by evaluating the pooled data on Bt-maize pollen with control maize pollen data, also pooled.

## Results

### Survival

**Figure 1:**
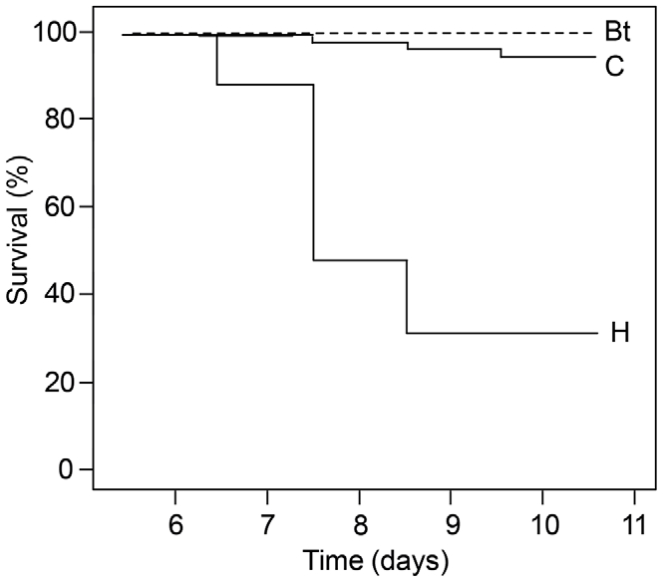
Survival analysis of honey bee larvae treated with pollen enriched diets. The dashed curve “Bt” indicates the 100% survival rate for Bt-pollen treated larvae (stacked Bt-maize expressing Cry1A.105, Cry2Ab2 and Cry3Bb1 and single Bt-maize expressing Cry1Ab were pooled; n = 40 larvae). Curve “C” indicates survival for three conventional (control) maize pollen treatments (pooled n = 59 larvae). No significant differences in survival rates were found among maize pollen treatments (neither individually, nor pooled). Compared to the other treatments, the larvae fed with the toxic *Heliconia rostrata* pollen (H; n = 10) had a significantly lowered survival rate.

All 40 larvae fed with Bt-maize pollen survived the 120 hours of dietary exposure upon the prepupae phase (Fig. 1). The survival rate of the conventional maize pollen fed larvae did not differ significantly from Bt-maize pollen fed larvae {C: 56 out of 59; 95%} (*Chisq* = 0.72, *df* = 1, *P* = 0.40). Of all the maize pollen fed larvae (N = 99), in total 97% survived until the prepupal phase. Specific survival rates were: for stacked Bt-maize 100%, near-isogenic line 100%, Mon810 100%, DKc4250 95%, and for Benicia 90%. Thus, no significant difference among the five maize pollen varieties was found (*Chisq* = 5.41, *df* = 4, *P* = 0.28).

Among the larvae fed with diets without pollen, the individual survival dynamics and the survival rate of 92% did not differ compared to larvae fed with maize pollen enriched diets (all *P* values≥0.64). In contrast, significantly fewer larvae survived the larval phase when they were fed with *H. rostrata* pollen compared to the other six treatments (*P* values≤0.01, all significant with an α/6 sequential Holm-Bonferoni correction) (Fig. 1).

### Sublethal effects on the prepupae weight

**Figure 2:**
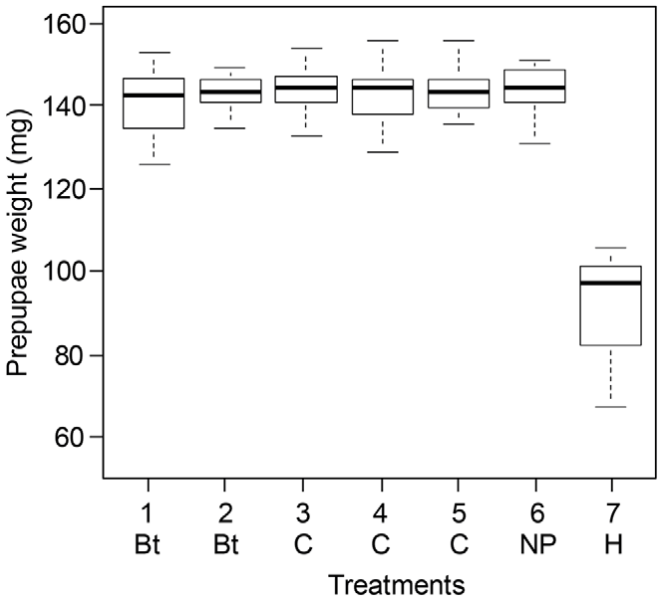
Prepupal weights (mg) of honey bee larvae fed with pollen. Treatments are Bt-maize pollen {Bt} (1 = stacked Bt-maize expressing Cry1A.105, Cry2Ab2 and Cry3Bb1; 2 = single Bt-maize expressing Cry1Ab) and non-GM maize pollen {C} (3 = near-isogenic line; 4 = distant related; 5 = unrelated) and two non-maize controls (6 = no pollen control {NP}; 7 = *Heliconia rostrata* {H}). The boxplots provide a graphical view of the median and quartiles with the error bars showing sample maximums and minimums. Prepupae weights did neither reveal a general Bt effect, nor single or stacked effects (GLMER: *P* values≥0.41). *H. rostrata* pollen fed larvae had significantly lower weights compared to all other treatments (GLMER: *P* values≤0.001).

With a mean of 142.3 mg, prepupae weights of Bt-maize pollen fed larvae were almost identical to the mean weight of conventional maize pollen fed larvae (142.6 mg; *t* = −0.20, *df* = 1, *P* = 0.82) (Table 2). A general variety-effect, considering possible differences between the five maize varieties, was not found (*F* = 0.26, *df* = 4, *P* = 0.90) thus the weight distributions of the transgenic and non-transgenic maize pollen treatments were all alike (Fig. 2).

**Table 2:** Prepupae numbers and weights after exposure to all individual dietary treatments, with a summarizing analysis for Bt-pollen (Bt) and non-GM pollen (C).

	Treatment	Prepupae weight Mean ± SD (n)	*P* values (GLMER with colony as random factor)						
			2	3	4	5	6	7	1,2
1	Stacked Bt maize	141.4±9.9 (20)	*0.42*	*0.41*	*0.90*	*0.88*	*0.86*	*<.0001* *	
2	Single Bt maize	143.3±4.9 (20)		*0.88*	*0.56*	*0.70*	*0.41*	*<.0001* *	
3	Near isogene (stacked)	143.5±4.9 (19)			*0.55*	*0.69*	*0.44*	*<.0001* *	
4	Distant related maize	142±10.5 (19)				*0.83*	*0.79*	*<.0001* *	
5	Unrelated maize	142.4±7.6 (18)					*0.64*	*<.0001* *	
6	No pollen	140.6±12.9 (11)						*0.0001* *	
7	H: *Heliconia rostrata*	87.7±21 (3)							
1,2	Bt: pooled Bt maize	142.3±7.7 (40)							
3,4,5	C: pooled control maize	142.6±9.1 (56)							*0.83*

*All *P* values are the results of paired tests: significances remain valid at the sequential Holm-Bonferoni correction of α/6 (considering the six comparisons per treatment).

Individual comparison shows that mean prepupae weights differed neither between stacked Bt-pollen and pollen from the near-isogenic line (*t* = 0.83, *df* = 33, *P* = 0.41), nor between the stacked Bt-variety and the single Bt-variety (*t* = 0.81, *df* = 34, *P* = 0.42) (Table 2). In contrast, *H. rostrata* pollen fed larvae showed a significantly lower mean prepupae weight compared to all the other treatments (mean 87.7 mg±21.0 SD; *P* values≤0.001) (Table 2).

## Discussion

Honey bees are the most important pollinators in agricultural ecosystems. In order to minimize the environmental risks of cultivating GM crops and their discussed contribution of being an underlying factor of the globally observed bee losses, robust and highly standardized risk assessment methods for honey bees are imperative. Here we present an effective pollen based method to test the direct effects of GM crops on *in vitro* reared larvae. Our test system reflects the natural exposure under field conditions and is therefore highly recommended for regulatory studies.

### Effects of pollen from single and multiple Bt-maize varieties on honey bee larvae

One recent trend in plant biotechnology is stacking of multiple insect resistance traits in a single cultivar [6]. Honey bees are exposed to mass flowering GM crops and not a single published study deals with the effect of stacked Bt-cultivars on bees. The results of this study did not indicate adverse effects of the consumption of single and stacked Bt-maize pollen on the survival and prepupae weight of *in vitro* reared *A. mellifera* larvae. At a realistic exposure dose, the 120 h survivorship of Bt-pollen treated larvae was 100% until the prepupae phase (Fig. 1). At the prepupae stage, where larvae had terminated feeding, digesting and growing, were no indications of a sublethal Bt-pollen effect on the weight of the prepupae (Fig. 2).

The outcome of our data on stacked Bt effects are in line with earlier brood tests under colony conditions on single insect resistant Bt-maize pollen [36] or single purified Bt-proteins [7], [37]. In contrast to these colony level studies, the current results are achieved by testing under controlled laboratory conditions, with minimum control mortality. Compared to single Bt-proteins in pollen or in purified form, our plant produced stacked Bt-proteins, with the chimeric Bt-protein Cry1A.105, indicate a similar level of safety. In accordance, a stacked maize variety, expressing Bt proteins VIP3A and Cry1Ab, also caused no adverse effects on the biodiversity of arthropods during a 3 year ERA field experiment [38]. A stacked cotton cultivar, expressing cowpea trypsin inhibitor (CpTI) and Cry1Ac in pollen carried no lethal risk for honey bees, though a worst case feeding regime did cause feeding inhibition [39]. However, in studies comparing Cry1 with transgenic protease inhibitors, it was found that only the latter was causing reduced feeding effects [12], [40], [41], [42].

The stacking of insect resistance traits in one crop aims to enhance the effectiveness towards target pest insects, to cause an additive or synergistic toxicity. Among target pest insects, synergistic effects between e.g. Cry1Ab, Cry1Ac, Cry1F and/or Cry2Ab2 have been reported [43], [44]. Involved in toxicant synergies are mostly uptake, transportation or degradation pathways [45], causing a higher toxicity and a lower selectivity. Hence, potential synergistic effects on non-target insect also deserve consideration. The honey bee, a key non-target insect, has never shown lethality to Bt-proteins [7] and our data support the notion that, synergistic effects by stacking Bt-proteins at plant produced levels are unlikely a risk to bees. However, sublethal effects [46] on feeding, learning performance and foraging behavior might occur [47], [48]. Indeed, the *in vitro* approach covers the opportunity of testing of potential sublethal effects, by a subsequent behavioral tests on hatched bees [49].

In order to examine a potential effect of increased protein expression levels, two Bt-maize varieties with different expression levels were compared. Bt-maize variety Mon89034×Mon88017 has compared to Mon810 a 10^2^ to 10^4^ times increased Bt-protein expression level in pollen (see material and methods). Hypothetically, Mon810 could have had Bt-protein levels under a toxic threshold, but the larvae remain unharmed by the multifold Bt-protein of stacked Bt-maize pollen.

### Pollen bioassays

The current bioassay tests GM plant material directly and realistically, by reflecting a natural consumption and digestion of pollen by *A. mellifera* larvae. It closes an important knowledge gap between *in vivo* colony experiments [7], [14], [36], [37], [38] and *in vitro* experiments with purified transgenic proteins [50], [51], [52]. Although purified proteins are ideal to test worst case exposure scenarios [9], [48], the *E. coli* produced purified Bt-substances do not represent a field situation. And although field experiments have realistic pollen exposure conditions, a down-side is a variety of uncontrolled environmental factors. In addition, pollinator field studies have to be synchronized to the flowering period and they are space and time consuming and therefore relatively costly [53]. Finally, within a bee colony many factors such as colony size, diseases, and nutrition could have an influence on the brood development. The presented robust bioassay minimizes any environmental effect on larval development and allows a good control of dietary pollen amounts (Table 1).

The conventional non GM maize cultivars (Table 1) allow a secure assessment of the impact of the introduced transgenic traits [54]. It makes assessments comprehensive, since it enables a reliable estimate of naturally occurring variation within the crop species. Though having tested the total of five maize varieties, no maize-sort related differences were found. Nevertheless, the toxic control treatment and the power analysis indicated that monitoring discernable effects of pollen on honey bee larvae was effective (Fig. S1, Fig. S2, Power analysis S1). At the given sample sizes, the test was able to distinguish effects on both the survival and the weight endpoint.

The functionality of the pollen bioassay is proven by the feeding dose of 1600 *H. rostrata* pollen, which caused significant lethal and sublethal effects on larvae. This dose caused 50% of the larvae to die in 72 hours (LT_50_) and 100% to die in 7 days (LT_100_). The results demonstrate the usefulness of positive controls in order to i) validate the ingestion of pollen treatments, ii) to demonstrate the capacity of detecting treatment effects and iii) to allow comparisons with other studies [9].

Precise and robust ERA methods are needed for honey bees [55]. Our bioassay is well suited to monitor environmental pollution of pollen or natural pollen toxicity (Pictures S1). Of genuine concern are systemic, lipophilic chemicals (e.g. neonicotinoids) as used in agriculture, because the plant pollen are a carrier of pesticides into honeybee colonies. Such pesticides may cause (sub-) lethal effects and can be extremely persistent [46]. Our pollen test is widely applicable and it fits international tiered risk assessment schemes for regulatory biosafety assessments of any new transgenic trait. Hence, we propose the *in vitro* bioassay for consideration as a standard pre-release test for all polleniferous transgenic crops.

## Supporting Information

Figure S1 **Statistical power analysis for survival data of honey bee larvae on maize pollen enriched diets.** The survival power analysis was based on a one-tailed 2-proportions test on mortality rate differences, comparing a control and a treatment group with a same sample size. Determining treatment effects more sensitively at higher sample sizes, the curves indicate the level of power with dotted lines for 0.4, striped lines for 0.6 and a continuous line for 0.8 power at analysis (significance level of α = 0.05). (Power analysis S1).
(TIF)

Figure S2 **Statistical power analysis for prepupae weight data of honey bee larvae on maize pollen enriched diets.** The weight difference power analysis was based on a two-tailed t-test on weight differences between the treatment group and the control (with same sample sizes). The sensitivity to measure the mg weight differences is relating to the general variance in weight of all maize pollen fed larvae (142 mg±8.5 SD, n = 96). The significance level of α = 0.05 at 0.4, 0.6 and 0.8 power determined which sample sizes were needed to indicate effects. (Power analysis S1).
(TIF)

Pictures S1 **A honey bee larvae** ***in vitro*** **bioassay for testing pollen toxicity, considering GM-maize pollen (** ***Zea mays*** **) and pollen of** ***Heliconia rostrata*** **.** Pictures by Harmen P. Hendriksma (legends are embedded in the pictures).
(PDF)

Power Analysis S1 **An analysis of statistical power to indicate mortality and weight differences within the experimental data.** Supplementary information in addition to Figure S1 and Figure S2 on power analysis.
(DOC)

## References

[1] Klein AM, Vaissière BE, Cane JH, Steffan-Dewenter I, Cunningham SA (2007). Importance of pollinators in changing landscapes for world crops.. Proceedings of the Royal Society of London Series B - Biological Sciences.

[2] Potts SG, Biesmeijer JC, Kremen C, Neumann P, Schweiger O (2010). Global pollinator declines: trends, impacts and drivers.. Trends in Ecology and Evolution.

[3] Gallai N, Salles JM, Settele J, Vaissière BE (2009). Economic valuation of the vulnerability of world agriculture confronted with pollinator decline.. Ecological Economics.

[4] Free JB (1993). Insect pollination of crops. 2^nd^ ed.

[5] Ruttner F (1988). Biogeography and Taxonomy of Honey Bees.

[6] James C (2010). Global Status of Commercialized Biotech/GM Crops: 2009. ISAAA Brief No. 42.

[7] Duan JJ, Marvier M, Huesing J, Dively G, Huang ZY (2008). A Meta-Analysis of Effects of Bt Crops on Honey Bees (Hymenoptera: Apidae).. PLoS ONE.

[8] Malone LA, Burgess EPJ, Ferry N, Gatehouse AMR (2009). Impact of Genetically Modified Crops on Pollinators.. Environmental Impact of Genetically Modified Crops.

[9] Romeis J, Hellmich RL, Candolfi MP, Carstens K, De Schrijver A (2011). Recommendations for the design of laboratory studies on arthropods for risk assessment of genetically engineered plants.. Transgenic Research.

[10] Brodschneider R, Crailsheim K (2010). Nutrition and health in honey bees.. Apidologie.

[11] Seeley TD (1985). Honey bee ecology.

[12] Babendreier D, Kalberer NM, Romeis J, Fluri P, Mulligan E (2005). Influence of Bt-transgenic pollen, Bt-toxin and protease inhibitor (SBTI) ingestion on development of the hypopharyngeal glands in honeybees.. Apidologie.

[13] Haydak MH (1970). Honey bee nutrition.. Annual Review of Entomology.

[14] Babendreier D, Kalberer N, Romeis J, Fluri P, Bigler F (2004). Pollen consumption in honey bee larvae: a step forward in the risk assessment of transgenic plants.. Apidologie.

[15] Felke M, Langenbruch GA (2005). Impact of transgenic Bt-maize pollen on larvae of selected butterflies.. http://www.bfn.de/fileadmin/MDB/documents/skript157.pdf.

[16] Glare TR, O'Callaghan M (2000). *Bacillus thuringiensis*: Biology, Ecology and Safety.

[17] Haydak MH (1970). Honey bee nutrition.. Annual Review of Entomology.

[18] Meissle M, Hellmich RL, Romeis J (2011). Impact of Cry3Bb1-expressing Bt maize on adults of the western corn rootworm, *Diabrotica virgifera virgifera* (Coleoptera: Chrysomelidae).. Pest Management Science.

[19] Vaughn T, Cavato T, Brar G, Coombe T, DeGooyer T (2005). A method of controlling corn rootworm feeding using a *Bacillus thuringiensis* protein expressed in transgenic maize.. Crop Science.

[20] Clark PL, Vaughn TYT, Meinke LJ, Molina-Ochoa J, Foster JE (2006). *Diabrotica virgifera virgifera* (Coleoptera: Chrysomelidae) larval feeding behaviour on transgenic maize (MON 863) and its isoline.. Journal of Economic Entomology.

[21] Malone LA, Pham-Delègue PH (2001). Effects of transgene products on honey bees (*Apis mellifera*) and bumblebees (*Bombus sp.*).. Apidologie.

[22] Aupinel P, Fortini D, Michaud B, Marolleau F, Tasei JN (2007). Toxicity of dimethoate and fenoxycarb to honey bee brood (*Apis mellifera*), using a new *in vitro* standardized feeding method.. Pest Management Science.

[23] Hendriksma HP, Härtel S, Steffan-Dewenter I (2011). Honey bee risk assessment: New approaches for *in vitro* larvae rearing and data analyses.. Methods in Ecology and Evolution.

[24] de Maagd RA, Bravo A, Crickmore N (2001). How *Bacillus thuringiensis* has evolved specific toxins to colonize the insect world.. Trends in Genetics.

[25] Nguyen HT, Jehle JA (2007). Quantitative analysis of the seasonal and tissue-specific expression of Cry1Ab in transgenic maize Mon810.. Journal of Plant Diseases and Protection.

[26] Miranda M (2008). Gene and DNA Sequence: *cry1A.105*.. http://bch.cbd.int/database/record-v4.shtml?documentid=43771.

[27] Auerbach MJ, Strong DR (1981). Nutritional ecology of *Heliconia* herbivores: experiments with plant fertilization and alternative hosts.. Ecological monographs.

[28] Simpson J (1955). The significance of the presence of pollen in the food of worker larvae of the honey-bee.. Quarterly Journal of Microscopical Science.

[29] Jung-Hoffmann I (1966). Die Determination von Königin und Arbeiterin der Honigbiene.. Z Bienenforsch.

[30] R Development Core Team (2010). R: A language and environment for statistical computing.. http://www.R-project.org.

[31] Pinheiro J, Bates D, DebRoy S, Sarkar D, the R Core team (2009). nlme: Linear and Nonlinear Mixed Effects Models.. http://cran.r-project.org/web/packages/nlme/index.html.

[32] Fox J (2002). An R and S-PLUS companion to applied regression.

[33] Ripley RM, Harris AL, Tarassenko L (2004). Non-linear survival analysis using neural networks.. Statistics in Medicine.

[34] Therneau T, Lumley T (2009). survival: Survival analysis, including penalised likelihood.. http://CRAN.R-project.org/package=survival.

[35] Holm S (1979). A simple sequentially rejective multiple test procedure.. Scandinavian Journal of Statistics.

[36] Hanley AV, Huang ZY, Pett WL (2003). Effects of dietary transgenic Bt corn pollen on larvae of *Apis mellifera* and *Galleria mellonella*.. Journal of Apicultural Research.

[37] Arpaia S (1996). Ecological impact of Bt-transgenic plants: 1. Assessing possible effects of CryIIIB toxin on honey bee (*Apis mellifera* L.) colonies.. Journal of Genetics and Breeding.

[38] Dively GP (2005). Impact of Transgenic VIP3A×Cry1Ab Lepidopteran-resistant Field Corn on the Nontarget Arthropod Community.. Environmental Entomology.

[39] Han P, Niu CY, Lei CL, Cui JJ, Desneux N (2010). Quantification of toxins in a Cry1Ac+CpTI cotton cultivar and its potential effects on the honey bee *Apis mellifera* L.. Ecotoxicology.

[40] Malone LA, Burgess EPJ, Gatehouse HS, Voisey CR, Tregidga EL (2001). Effects of ingestion of a *Bacillus thuringiensis* toxin and a trypsin inhibitor on honey bee flight activity and longevity.. Apidologie.

[41] Sagili RR, Pankiw T, Zhu-Salzman K (1995). Effects of soybean trypsin inhibitor on hypopharyngeal gland protein content, total midgut protease activity and survival of the honey bee (*Apis mellifera* L.).. Journal of Insect Physiology.

[42] Babendreier D, Reichhart B, Romeis J, Bigler F (2008). Impact of insecticidal proteins expressed in transgenic plants on bumblebee microcolonies.. Entomologia experimentalis et applicata.

[43] Lee MK, Curtiss A, Alcantara E, Dean DH (1996). Synergistic Effect of the *Bacillus thuringiensis* Toxins CryIAa and CryIAc on the Gypsy Moth, *Lymantria dispar*.. Applied and Environmental Microbiology.

[44] Sharma P, Nain V, Lakhanpaul S, Kumar PA (2010). Synergistic activity between *Bacillus thuringiensis* Cry1Ab and Cry1Ac toxins against maize stem borer (*Chilo partellus* Swinhoe).. Letters in Applied Microbiology.

[45] Andersen ME, Dennison JE (2004). Mechanistic approaches for mixture risk assessments – present capabilities with simple mixtures and future directions.. Environmental Toxicology and Pharmacology.

[46] Desneux N, Decourtye A, Delpuech JM (2007). The sublethal effects of pesticides on beneficial arthropods.. Annual Review of Entomology.

[47] Ramirez-Romero R, Chaufaux J, Pham-Delegue MH (2005). Effects of Cry1Ab protoxin, deltamethrin and imidacloprid on the foraging activity and the learning performances of the honeybee *Apis mellifera*, a comparative approach.. Apidologie.

[48] Ramirez-Romero R, Desneux N, Decourtye A, Chaffiol A, Pham-Delegue MH (2008). Does CrylAb protein affect learning performances of the honey bee *Apis mellifera* L. (Hymenoptera, Apidae)?. Ecotoxicology and Environmental Safety.

[49] Brodschneider R, Riessberger-Gallé U, Crailsheim K (2009). Flight performance of artificially reared honeybees (*Apis mellifera*).. Apidologie.

[50] Malone LA, Tregidga EL, Todd JH, Burgess EPJ, Philip BA (2002). Effects of ingestion of a biotin-binding protein on adult and larval honey bees.. Apidologie.

[51] Brødsgaard HF, Brødsgaard CJ, Hansen H, Lovei GL (2003). Environmental risk assessment of transgene products using honey bee (*Apis mellifera*) larvae.. Apidologie.

[52] Lima MAP, Pires CSS, Guedes RNC, Nakasu EYT, Lara MS (2011). Does Cry1Ac Bt-toxin impair development of worker larvae of Africanized honey bee?. Journal of Applied Entomology.

[53] Romeis J, Bartsch D, Bigler F, Candolfi MP, Gielkens MMC (2008). Assessment of risk of insect-resistant transgenic crops to nontarget arthropods.. Nature Biotechnology.

[54] Rauschen S, Schultheis E, Pagel-Wieder S, Schuphan I, Eber S (2009). Impact of Bt-corn Mon88017 in comparison to three conventional lines on *Trigonotylus caelestialium* (Kirkaldy) (Heteroptera: Miridae) field densities.. Transgenic Research.

[55] Hendriksma HP, Härtel S (2010). A simple trap to measure worker bee mortality in small test colonies.. Journal of Apicultural Research.

