# Supplementary figures and images for: Testing Pollen of Single and Stacked Insect-Resistant Bt-Maize on In vitro Reared Honey Bee Larvae
Source: PLoS One. 2011 Dec 16;6(12):e28174. doi: 10.1371/journal.pone.0028174 (PMC3241620; doi:10.1371/journal.pone.0028174)

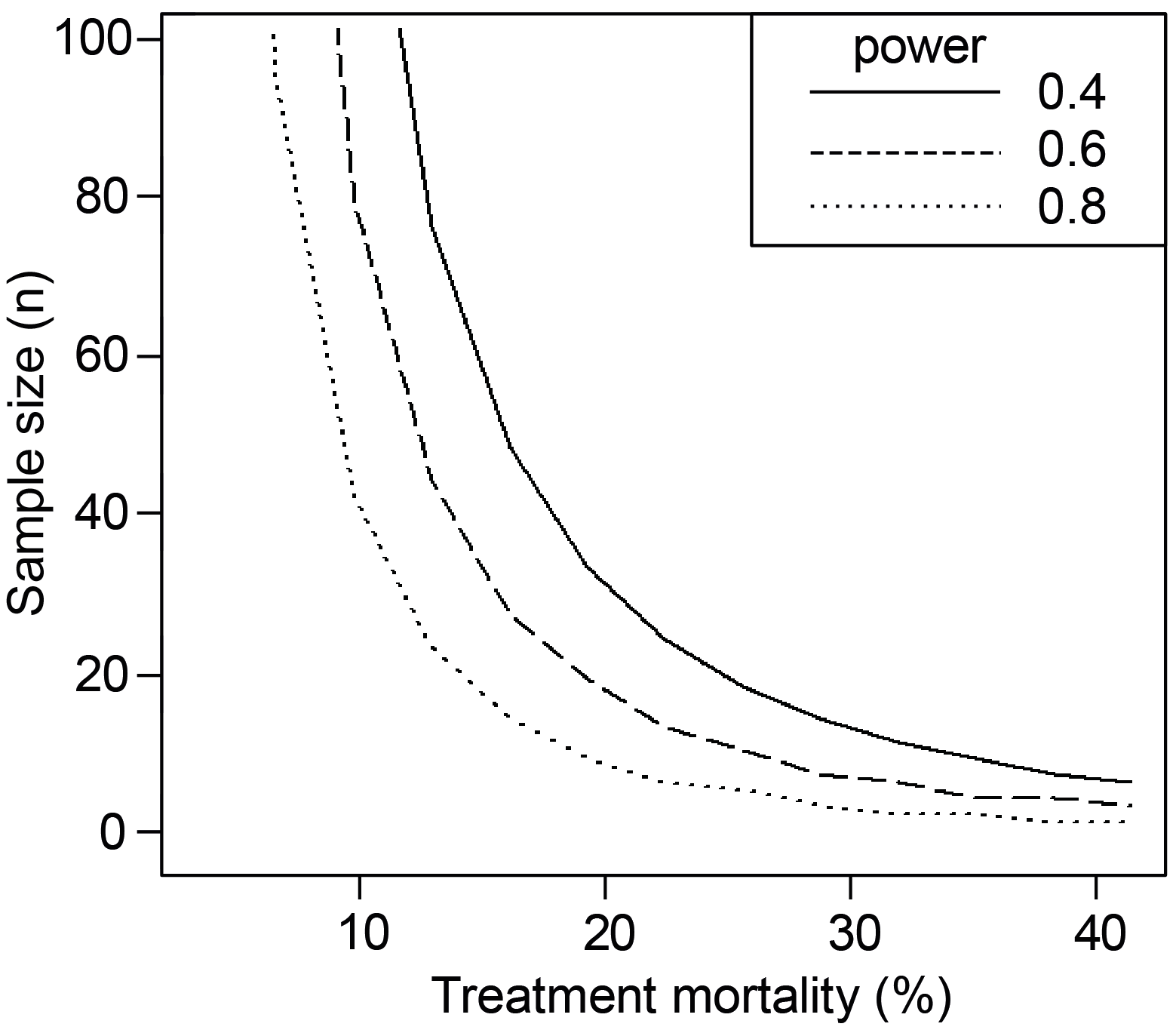

Supplement: Figure S1 — Statistical power analysis for survival data of honey bee larvae on maize pollen enriched diets. The survival power analysis was based on a one-tailed 2-proportions test on mortality rate differences, comparing a control and a treatment group with a same sample size. Determining treatment effects more sensitively at higher sample sizes, the curves indicate the level of power with dotted lines for 0.4, striped lines for 0.6 and a continuous line for 0.8 power at analysis (significance level of α = 0.05). (Power analysis S1). (TIF) [file pone.0028174.s001.tif]

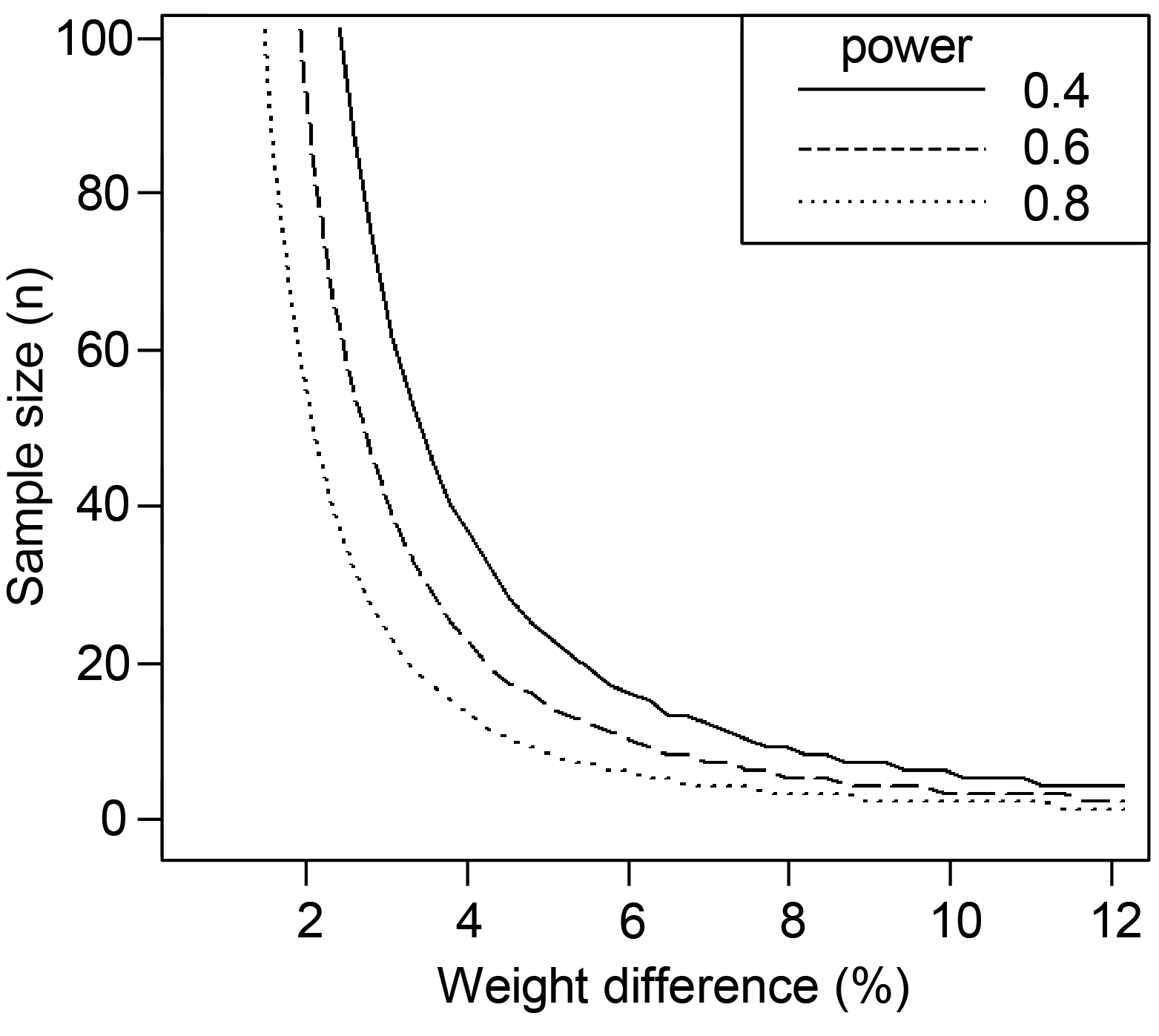

Supplement: Figure S2 — Statistical power analysis for prepupae weight data of honey bee larvae on maize pollen enriched diets. The weight difference power analysis was based on a two-tailed t-test on weight differences between the treatment group and the control (with same sample sizes). The sensitivity to measure the mg weight differences is relating to the general variance in weight of all maize pollen fed larvae (142 mg±8.5 SD, n = 96). The significance level of α = 0.05 at 0.4, 0.6 and 0.8 power determined which sample sizes were needed to indicate effects. (Power analysis S1). (TIF) [file pone.0028174.s002.tif]
