# Supplementary material for: Testing Pollen of Single and Stacked Insect-Resistant Bt-Maize on In vitro Reared Honey Bee Larvae
Source: PLoS One. 2011 Dec 16;6(12):e28174. doi: 10.1371/journal.pone.0028174 (PMC3241620; doi:10.1371/journal.pone.0028174)
Supplement: Pictures S1 — A honey bee larvae in vitro bioassay for testing pollen toxicity, considering GM-maize pollen ( Zea mays ) and pollen of Heliconia rostrata . Pictures by Harmen P. Hendriksma (legends are embedded in the pictures). (PDF) [file pone.0028174.s003.pdf]

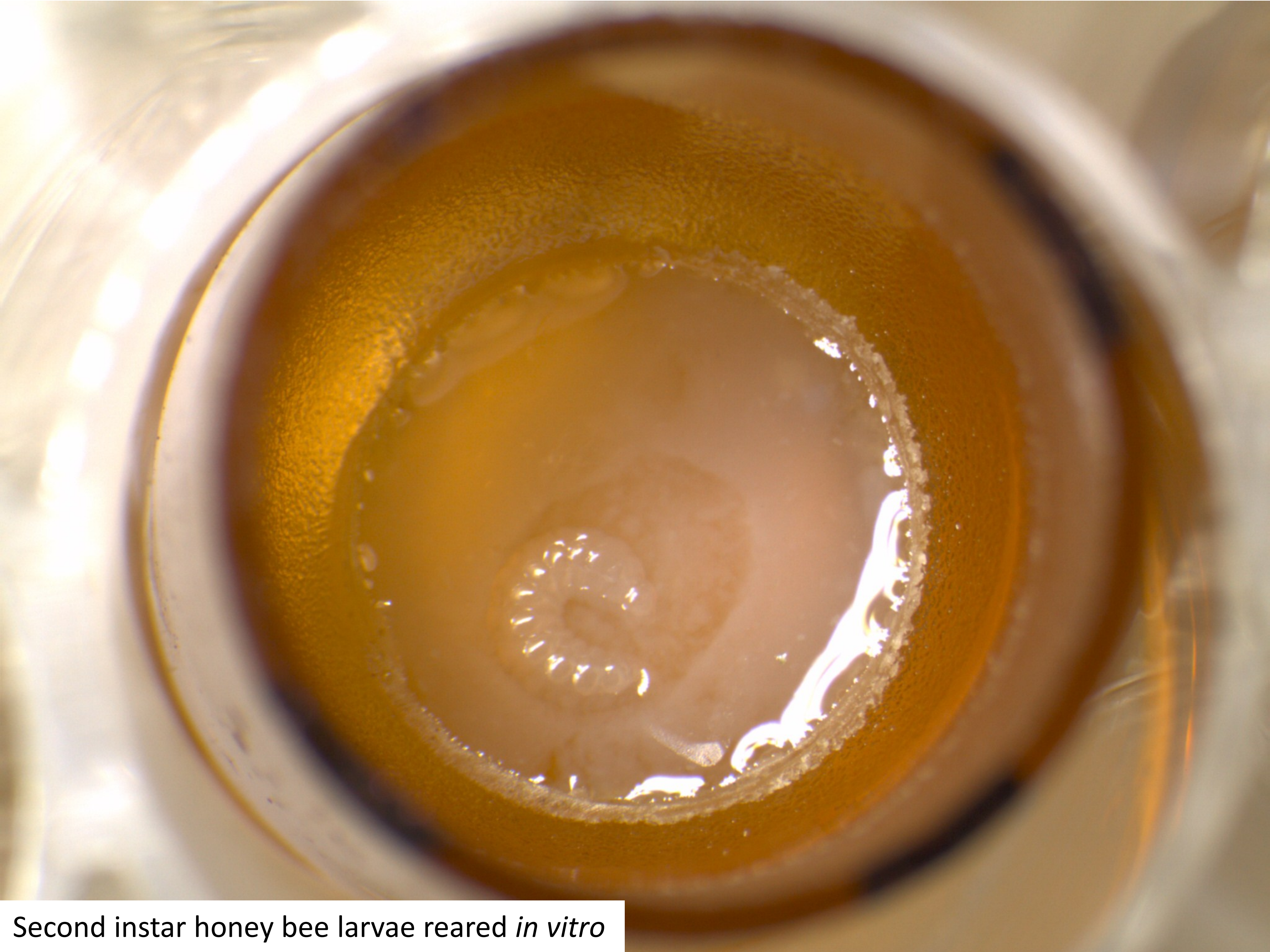

Second instar honey bee larvae reared *in vitro*

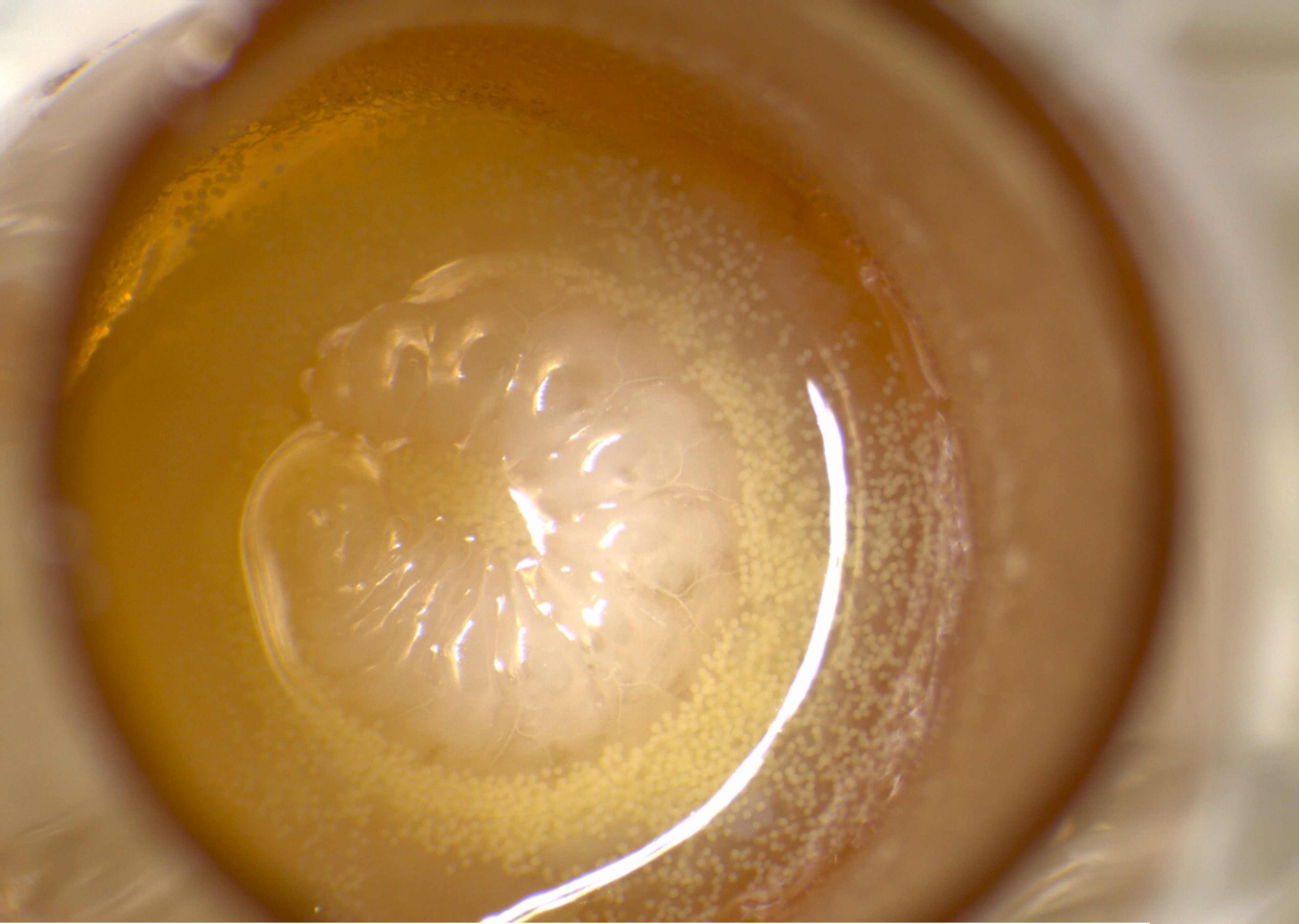

Forth instar honey bee larvae, feeding on maize pollen within the semi-artificial diet

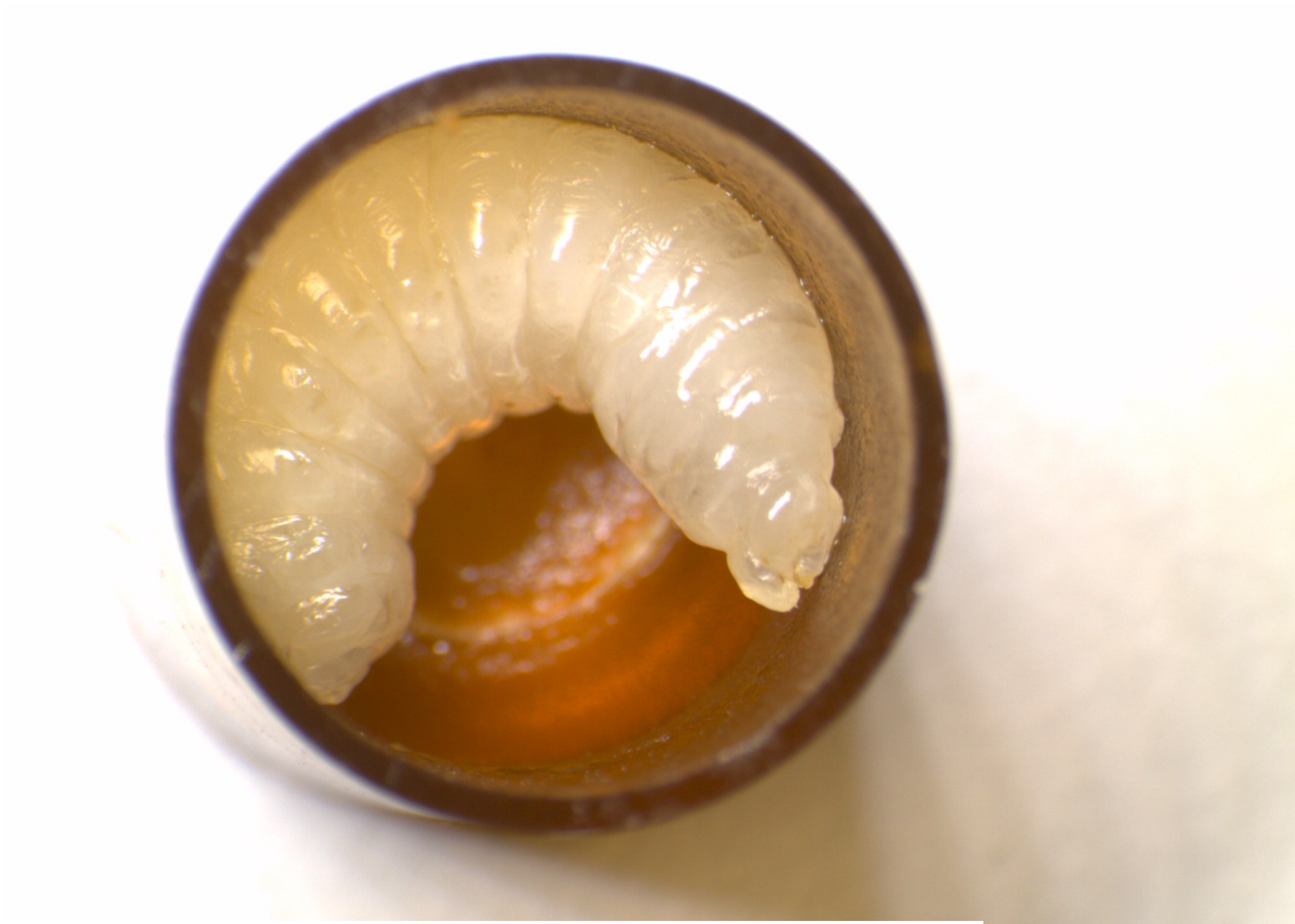

Larva finished feeding; Prepupal stage. Endpoint: weight and survival

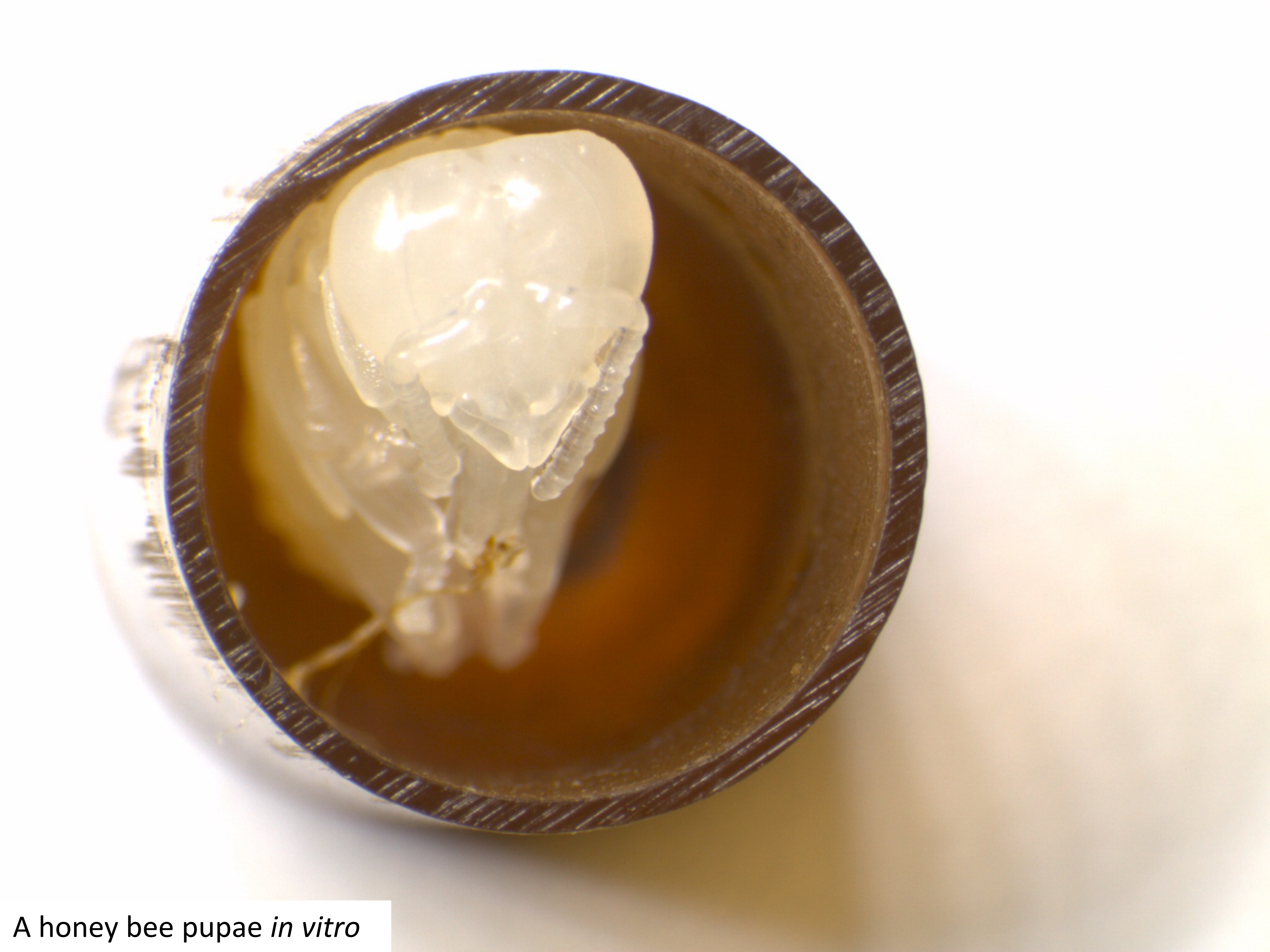

A honey bee pupae *in vitro*

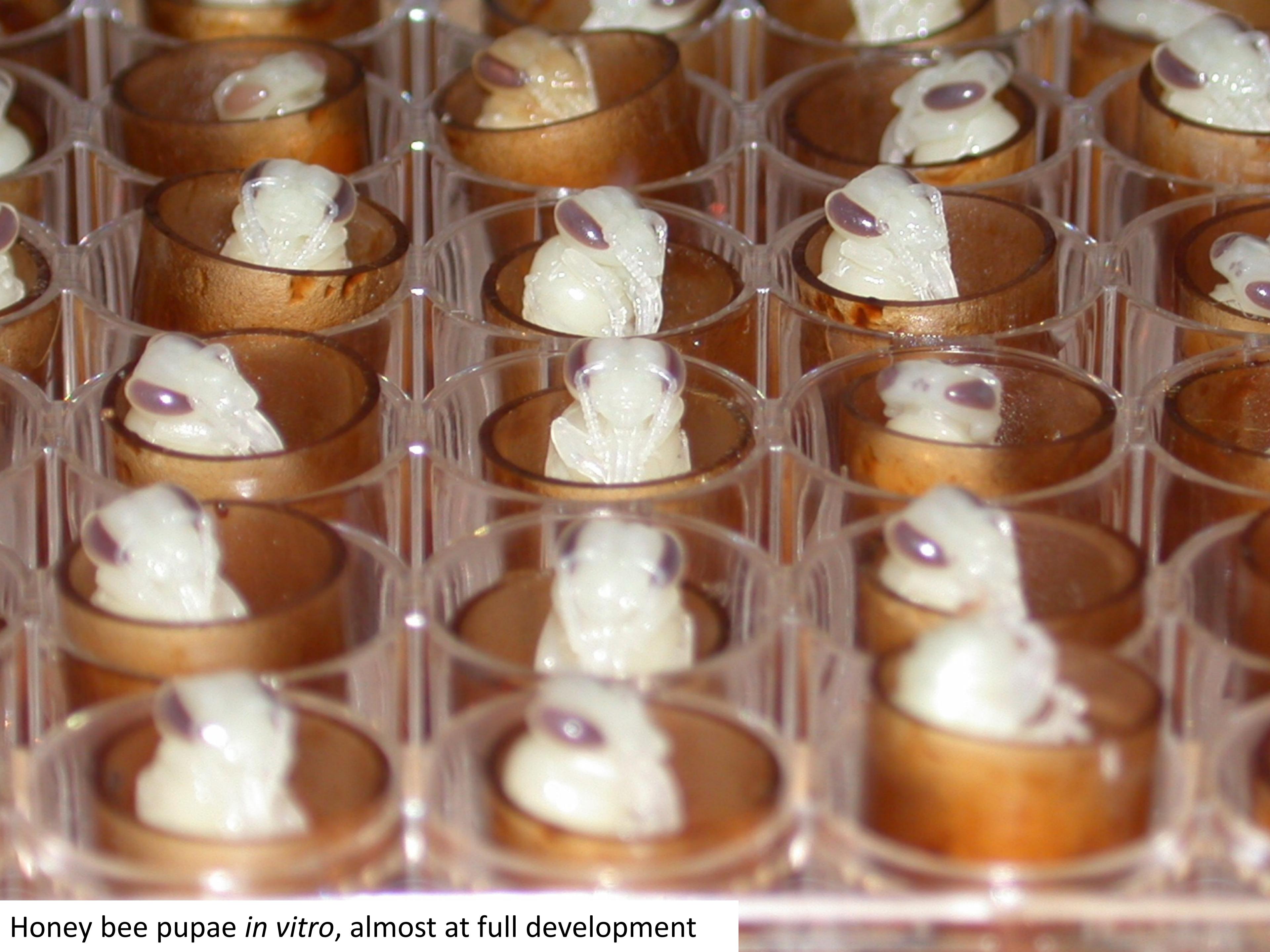

Honey bee pupae *in vitro*, almost at full development

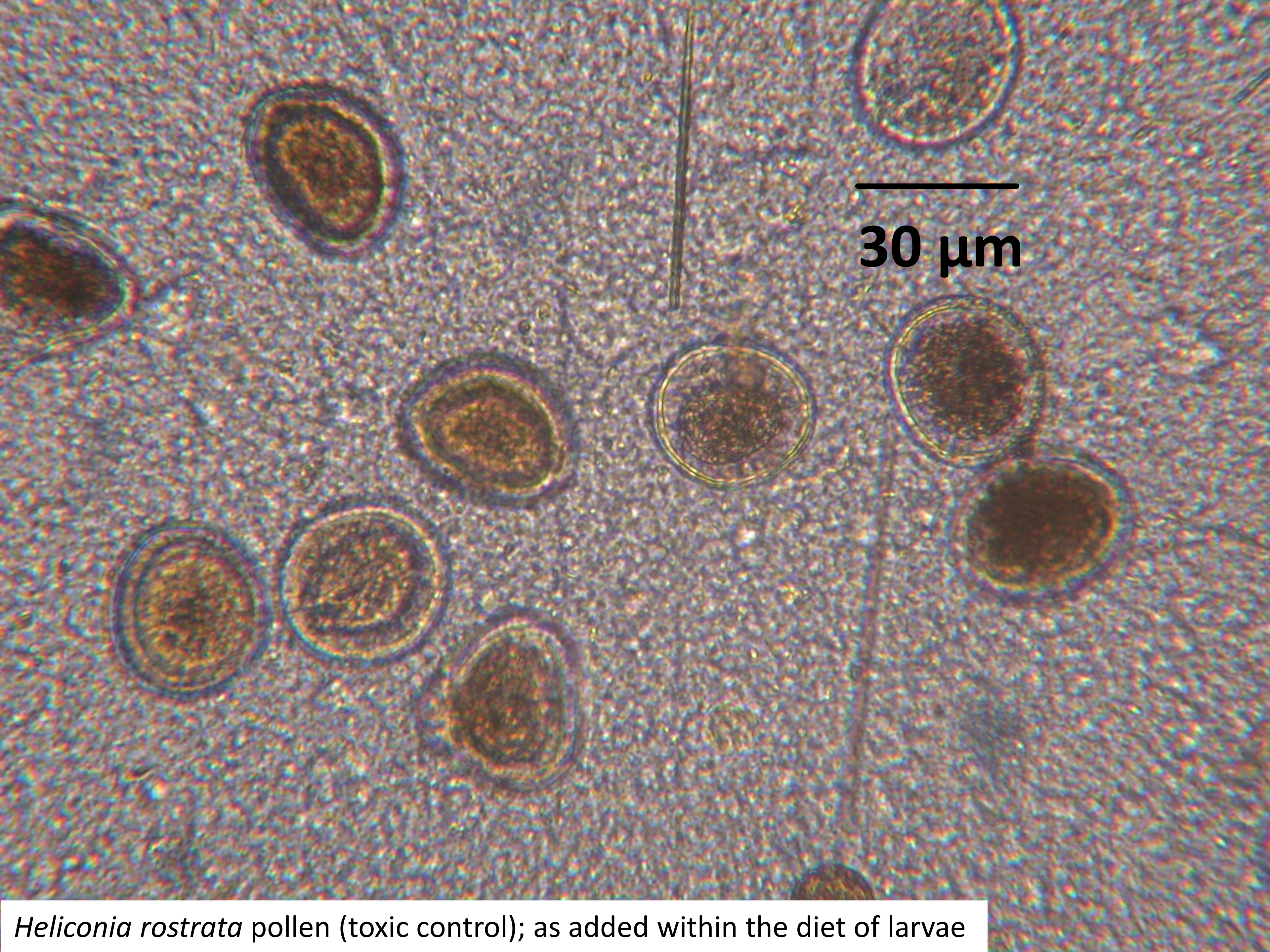

Heliconia rostrata pollen (toxic control); as added within the diet of larvae

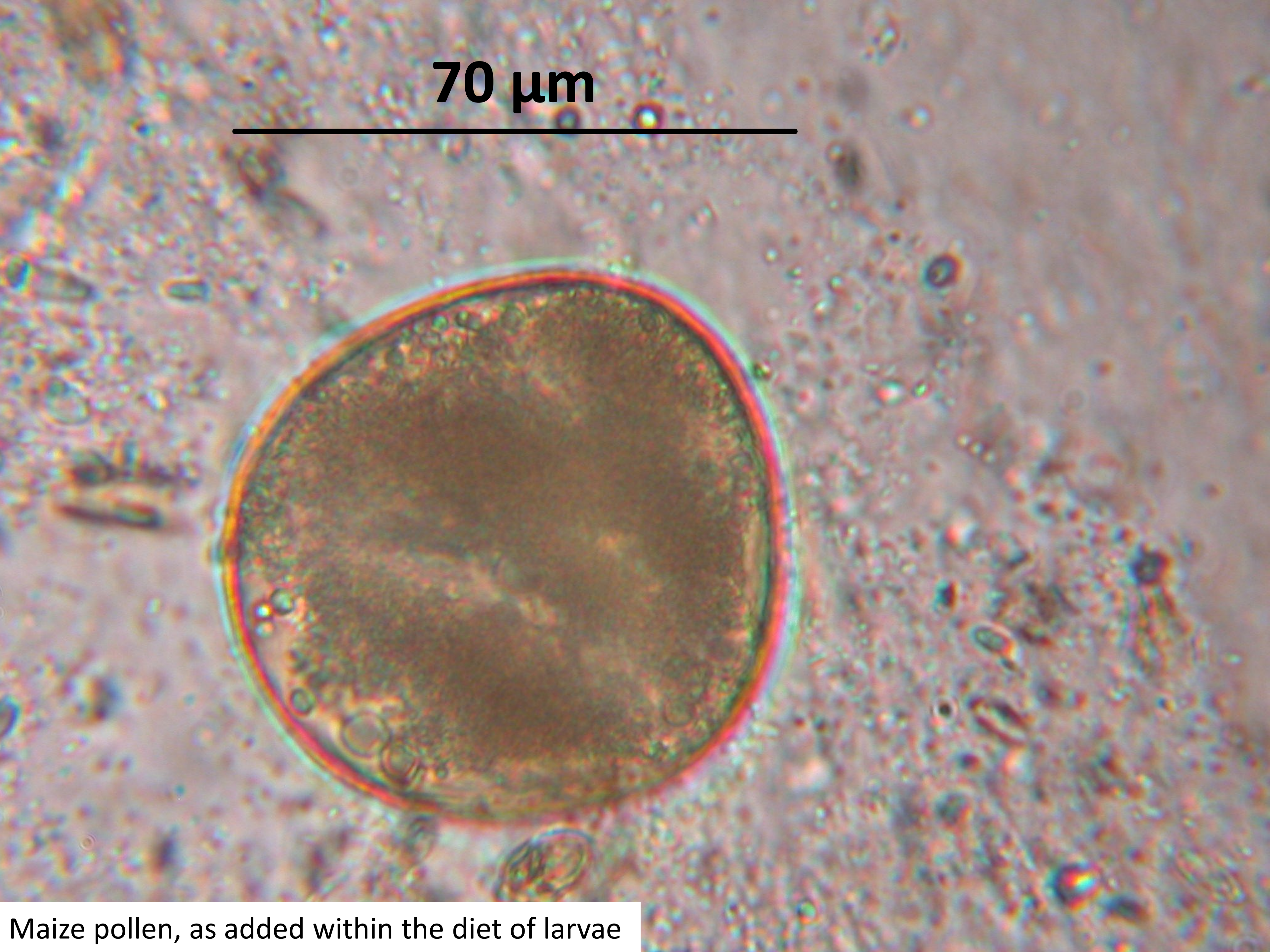

Maize pollen, as added within the diet of larvae

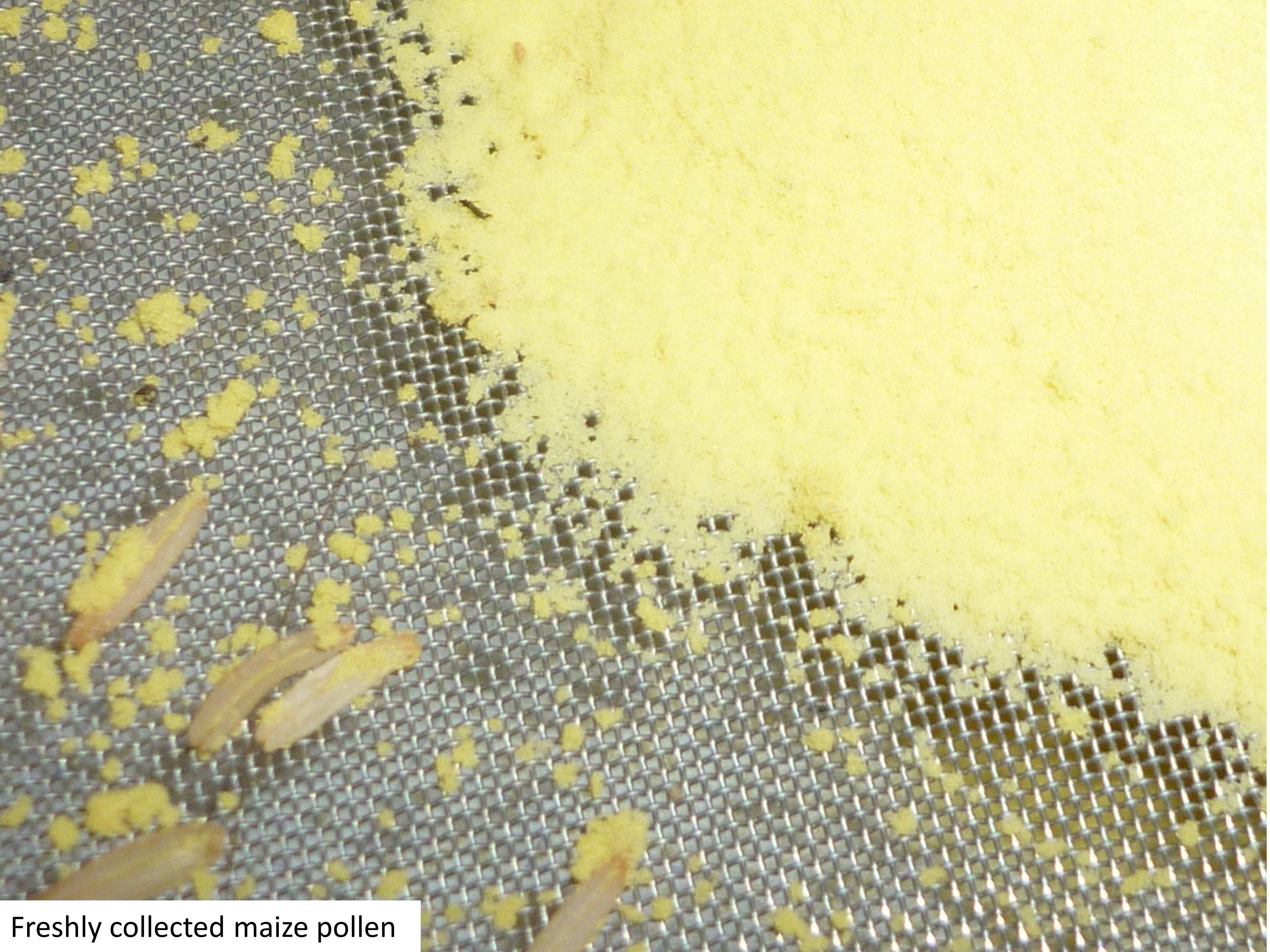

Freshly collected maize pollen
