# Supplementary material for: Testing Pollen of Single and Stacked Insect-Resistant Bt-Maize on In vitro Reared Honey Bee Larvae
Source: PLoS One. 2011 Dec 16;6(12):e28174. doi: 10.1371/journal.pone.0028174 (PMC3241620; doi:10.1371/journal.pone.0028174)
Supplement: Power Analysis S1 — An analysis of statistical power to indicate mortality and weight differences within the experimental data. Supplementary information in addition to Figure S1 and Figure S2 on power analysis. (DOC) [file pone.0028174.s004.doc]

**S4: Supplementary text to Fig. S1 and Fig. S2**

**POWER ANALYSIS**

Statistical power was determined with the *pwr* package of the R software (Champely 2009). A significance level of α=0.05 at 0.4, 0.6 and 0.8 power, determined which sample sizes were needed to indicate treatment effects on honey bee larvae. The mixed effect models with multi-comparisons, random effects and unequal sample sizes are represented by 2 power tests at a basic level by assuming a single comparison between a control and a treatment group with a same sample size. The survival power analysis was based on comparing a 100% survival of controls (proportion p_0_ = 1.0) with a reduced treatment survival (p_1_ ≤ 1.0) using a one-tailed 2-proportions test. Accordingly, considering our empirically determined variance in prepupae weight data, a two-tailed t-test was used for power analysis on the determination of weight differences.

Considering the *H. rostrata*, the single maize pollen and the pooled Bt-maize treatment; with 0.8 power, sample sizes of 10 20 and 40 larvae can be used to indicate mortality effects >35%, >25% and >18% respectively (Fig S1). Considering all maize pollen fed larvae (142 mg ± 8.5 SD, n=96), the 0.14% prepupae weight difference between the pooled Bt-maize and the controls maize treatments (Table 2; Bt *vs*. C) would need a sample size of >10.000 individuals for a significant result (α=0.05, 0.8 power). The bioassays’ detection of 5.4% prepupae weight difference with 0.8 power at a sample size of 20 larvae per group, shows good prospects for indicating possible sublethal effects (Fig. S2).

Champely S (2009). pwr: Basic functions for power analysis. R package version 1.1.1. http://CRAN.R-project.org/package=pwr (Accessed 27 JUL 2011)
